# Apremilast Cocrystals with Phenolic Coformers

**DOI:** 10.3390/molecules29246060

**Published:** 2024-12-23

**Authors:** Yelizaveta Naumkina, Bohumil Kratochvíl, Elena Korotkova, Jan Čejka

**Affiliations:** 1 Department of Solid State Chemistry, University of Chemistry and Technology, Prague, Technicka 5, 16628 Prague, Czech Republic; naumkiny@vscht.cz (Y.N.); bohumil.kratochvil@vscht.cz (B.K.); 2 Chemical Engineering Department, National Research Tomsk Polytechnic University, Lenin Avenue 30, 634050 Tomsk, Russia; eikor@tpu.ru

**Keywords:** cocrystals, Apremilast, phenolic compounds, π–π interactions, hydrogen bonds

## Abstract

Apremilast (APR) is an anti-inflammatory drug commonly used in the treatment of psoriasis. In efforts to enhance its solubility, several cocrystals with similar structural features have been developed. This study investigates the cocrystallization of APR with four phenolic-type coformers: phenol, catechol, pyrogallol, and hydroxyquinol. These coformers differ in the number and position of their hydroxyl groups, with their melting points varying by as much as 100 °C. Four novel cocrystal forms were synthesized, purified, and characterized using X-Ray diffraction and thermal analysis techniques. Surprisingly, the resulting cocrystals exhibited minimal differences in their melting points. The molecular packing of APR appears to limit the network-forming potential of the hydroxyl groups, a conclusion supported by the solved crystal structures, Hirshfeld surface analysis, and differential scanning calorimetry (DSC) results.

## 1. Introduction

Crystal engineering studies the design and properties of crystalline solid forms and their applications. It provides new perspectives for the materials design [1]. By understanding the intermolecular bonds and their hierarchical nature, their bonding, and orientations, it is possible to modulate the solid-phase crystal structure. Multicomponent crystals, such as solvates, salts, and cocrystals are the subjects of study in crystal engineering. Pharmaceutical cocrystals are particularly attractive because their composition can enhance the properties of the active pharmaceutical ingredient (API) [2]. The pharmaceutical cocrystals are solid crystalline single-phase materials comprising two or more neutral components (API and a guest molecule) in stoichiometric ratio, bound by non-covalent bonds such as hydrogen bonding, weak π–π stacking, and van der Waals forces. The cocrystals enable API properties to be changed without altering the API chemical structure. Also, the cocrystal design is open to more complicated forms like cocrystals of salts, solvated cocrystals, or solvated cocrystals of salts [3].

Pharmaceutical cocrystal development is used with the aim of modifying both physicochemical and pharmacokinetic properties. At the preliminary development stage, the target pharmaceutical drug molecules and coformers (small organic compounds) are selected and studied to determine whether it is possible to form a stable cocrystal [2]. A coformer is usually a small organic molecule (not a solvent) of appropriate size and shape for solid-state interactions with the API in the crystal lattice. The coformer is desired to be soluble in the solvent or the medium of interest, thermally and chemically stable and cost-effective. The CCDC database is a convenient database for selecting and analyzing potential coformers’ structures for the possibility of non-covalent interactions [4].

Apremilast (APR), a non-steroidal drug used to treat inflammatory conditions such as psoriasis and psoriatic arthritis, was studied. The pathogenesis of psoriatic plaques involves the active involvement of keratinocytes and neutrophils, which secrete the enzyme PDE4 [5]. Inhibiting PDE4 has been identified as a feasible strategy to promote immune balance and suppress the inflammatory response. Among the various PDE4 inhibitors used for the treatment of dermatological conditions, APR stands out as a prominent example [6].

Due to its low solubility and low permeability, APR is classified as a Class IV drug in the Biopharmaceutical Classification System (BCS). It has been published that the oral bioavailability of the API ranges from 20% to 33%, and its absolute bioavailability has been measured at roughly 73% [7]. Formulation development, particularly in liquid dosage forms, is made complex by its poor solubility in water [7]. Poor solubility of Apremilast as an oral drug is a serious issue that needs to be addressed [8].

Several solutions have been proposed to improve the solubility and bioavailability of Apremilast. To modify its bioavailability, amorphous solid dispersions of APR with various polymers were created. Pharmacokinetic studies of these dispersions showed an enhancement in bioavailability. However, a decrease in dissolution profiles for the dispersions was observed when stored under conditions of 75% relative humidity [8].

Hence, cocrystallization could improve poor properties without changing the chemical structure of the API [3]. Thirty-six multicomponent systems of APR involving various crystallization partners have been reported [9,10,11,12,13,14,15]. Solid forms crystallize in the tetragonal system with space group *P*4_1_2_1_2. All coformers used in the synthesis of APR cocrystals show structural similarity, and they are aromatic compounds. Also, both APR and guest molecules are bound preferably by aromatic–aromatic interactions. Even though APR and coformer molecules have strong hydrogen bond donors and acceptors, it remains unclear what changes are observed in the interactions in the structures of solid forms with several structurally identical coformers.

**Scheme 1:**
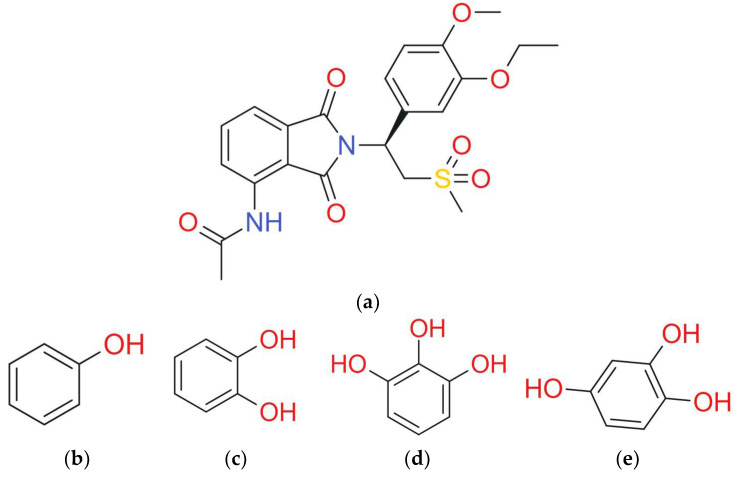
Apremilast (**a**) and coformer structures: (**b**) Phenol (PHE); (**c**) Catechol (CAT); (**d**) Pyrogallol (PYR); (**e**) Hydroxyquinol (HXQ).

Complementary to published studies, we introduced a series of phenolic compounds as structurally similar coformers. The idea behind this was to both broaden π–π stacking features and introduce hydrogen bond donors and acceptors, which could eventually help to develop forms with different molecular packing. The general formula of phenols is C_6_H_6-n_(OH)_n_. Cocrystallization was carried out with phenolic compounds (catechol (CAT), pyrogallol (PYR), phenol (PHE), hydroxyquinol (HXQ), and (Scheme 1) which are well known for their biological and environmental importance, antioxidation, and antiviral properties. Phenolic-type compounds such as catechol [16,17], resorcinol [18], hydroquinone [18], phloroglucinol [19], hydroxyquinol [16,17], and pyrogallol [20] have previously been studied as potential coformers with various APIs. Phenol, being a toxic compound, is not used in drug formulations. Recently, APR:CAT solid form was synthesized by mechanochemical grinding. The crystal structure was established using NMR crystallography approach [11].

## 2. Results and Discussion

### 2.1. Screening Results

New multicomponent forms of APR with phenolic compounds were studied. All previously reported crystal structures of Apremilast with phenolic compounds tend to crystallize in the tetragonal space group *P*4_1_2_1_2 [9,10,12,13,14,15]. Apremilast molecules construct a rigid framework that encloses the guest molecule in a cavity. Several phenolic compounds were selected. We expected that the positions of the functional group in the guest molecule could significantly influence the size and shape of the cavity and hydrogen bond network.

### 2.2. Crystal Structures

**Figure 1:**
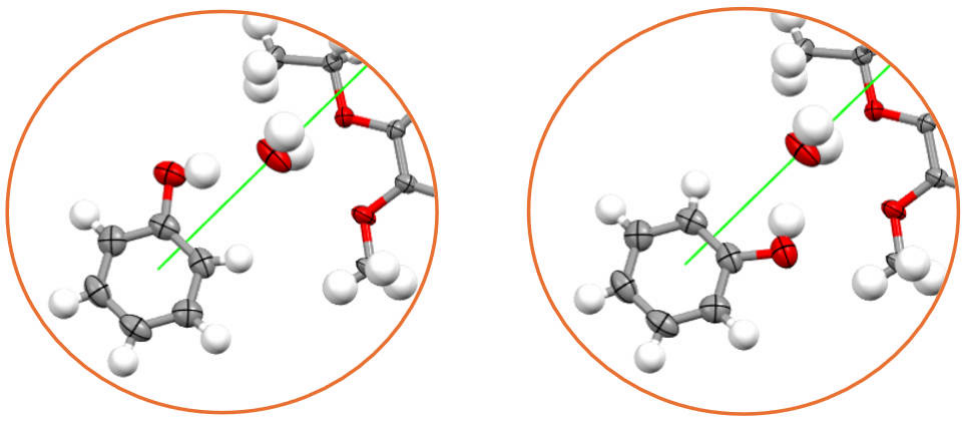
PHE coformer position on the two-folded axes in the APR:PHE cocrystal.

The hemihydrate cocrystals APR:PHE, APR:CAT, APR:PYR, APR:HXQ crystallize as a tetragonal bipyramid in the *P*4_1_2_1_2 space group. In each case, the asymmetric unit comprises one independent molecule of APR, a half molecule of each respective conformer, and a half molecule of water. The coformer and water molecule occupy a special position on the two-fold axis in the *z*-direction. Despite using slightly different crystallizing conditions (APR:HXQ was prepared by slow evaporation, while APR:PHE, APR:CAT, and APR:PYR were prepared by the grinding methods), crystal structures with barely the same unit cell parameters were obtained. As the coformer molecule resides in the special position, the atom positions are disordered by rotation around the two-fold axis. In the graphical representation of the model, it appears just like there is a partial molecular disorder of the hydroxyl groups in the benzene ring with an occupancy of 50%. The two-fold rotation axis passing through the benzene ring of the coformer describes this situation (Figure 1). All attempts to solve the structures in lower symmetry *P*4_1_, even *P*1, to break the disorder resulted in the disordered coformer molecule. Structures of the hemihydrate cocrystals are described and compared together since they have almost similar intermolecular interactions.

Discussed hydrogen bond distances and geometry of the structures are presented in Table 1 (All hydrogen bond distances and parameters are presented in Table S1). The first and most interesting characteristic of the structures is the presence of water molecule. The water molecule is located on the two-fold axis. It forms a bridge between APR and the coformer. Water binds with the alkoxy group of APR. The hydrogen bond distance between the water and alkoxy group of APR (O50–H···O6) is 2.993(2) Å, 2.929(3) Å, 2.883(4) Å, and 2.952(4) Å, respectively, in structures APR:PHE—APR:CAT—APR:PYR—APR:HXQ (Table 1). It can be noticed that the hydrogen bond shortens as the number of hydroxyl groups in the ring increases. The hydrogen bond distance between water and hydroxyl group of coformer (O47–H···O50) is 2.713(4) Å, 2.609(4) Å, 2.676(4) Å, and 2.515(6) Å, respectively, in the row APR:PHE—APR:CAT—APR:PYR—APR:HXQ (Table 1). Here, it can also be observed that the bond length decreases (APR:PHE—APR:CAT—APR:HXQ) both with the increase in the number of substituents and with the change of the position of substituents in the ring (APR:PYR—APR:HXQ).

In crystal structures of APR:PYR and APR:HXQ in coformers, the hydroxyl group (O48–H481) does not form any interactions with the surrounding molecules due to the distant position of these molecules.

**Figure 2:**
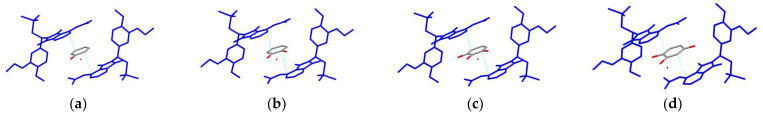
Tetrameric motifs of structures formed by π–π interactions: (**a**) APR: PHE; (**b**) APR: CAT; (**c**) APR: PYR and (**d**) APR: HXQ. APR molecules in blue color for clarity.

Coformers and APR molecules are bonded with π–π interaction (Figure 2). The phthalimide ring plane (C1C2C3C4C5C6N1C7C8) of APR and coformer ring (C41C42C43′C43C42C41′), symmetry code: [*y*, *x*, 1 − *z*] are almost parallel with distances as 4.1187(11) Å, 4.1301(19) Å, 4.1255(17) Å, and 4.127(2) Å in the order APR:PHE, APR:CAT, APR:PYR, and APR:HXQ, respectively (Table 1). The dihedral angle made by planes of phthalimide and benzene ring is 5.35(9)°, 4.81(15)°, 4.40(13)°, and 5.81(18)°, respectively. With an increasing number of the -OH groups on the benzene ring of the coformer, the dihedral angle gets slightly sharper, except for APR:HXQ. Distinct dihedral angle (<(O50–H···O6) = 5.81(18)°) in the APR:HXQ is linked to OH group position in 1,3,4 in the benzene ring of HXQ.

### 2.3. Structure Packing Similarity

**Figure 3:**
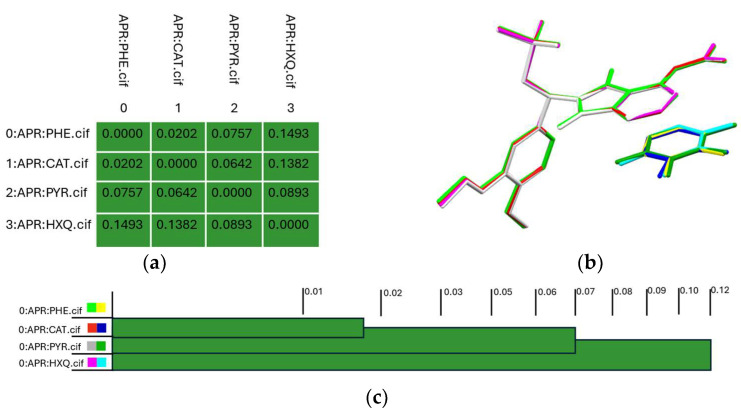
(**a**) PS*_ab_* value matrix for the pairs of APR solid forms; (**b**) APR solid forms overlapping (see Similarity dendrogram for used colors); (**c**) Similarity dendrogram of the molecular clusters of APR solid forms.

The similarity estimation of the solved crystal structures was performed using the software CrystalCMP rev.111 [21]. CrystalCMP offers a comparative analysis of molecular packing comparison by contrasting the molecular clusters in the dendrogram. According to packing similarity parameter’s low values, PS*_ab_* structures are in good agreement and distinguish slightly. The structure of APR:PHE is quite similar to the APR:CAT (PS*_ab_*01 = 0.0202). Further, the matching of APR:PHE with other crystal structures gradually decreases. However, the structure of APR: HXQ showed the slightest similarity with APR:PHE (PS*_ab_*03 = 0.1493), APR:CAT (PS*_ab_*13 = 0.1382), APR:HXQ (PS*_ab_*23 = 0.0893), which can be attributed to a change in the number and the substituent position in the benzene ring of coformer (Figure 3).

### 2.4. Powder X-Ray Diffraction (PXRD)

**Figure 4:**
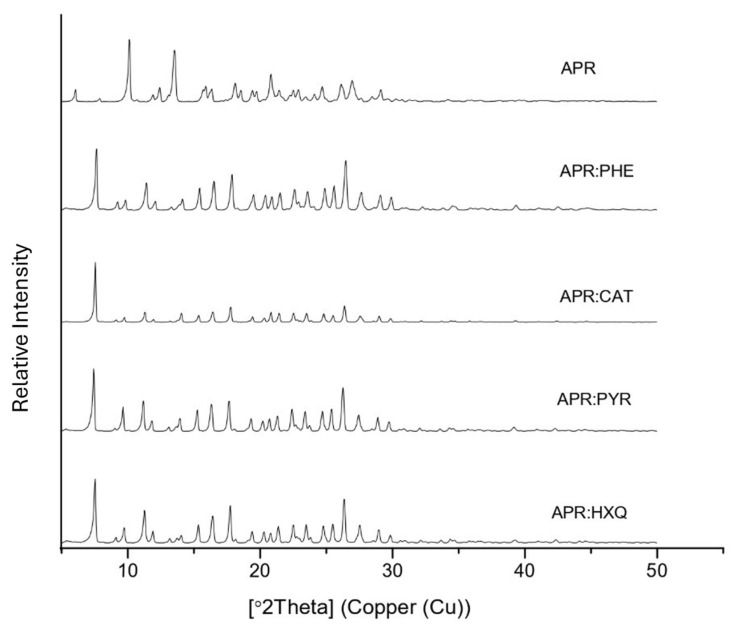
PXRD patterns of the Apremilast and new solid forms.

The new phases obtained were rinsed with acetone to remove the reagents. Afterwards, the resulting phases were characterized by the powder X-Ray diffraction method (PXRD) to confirm their purity. The diffraction patterns of the APR hemihydrate cocrystals were found to be very close to each other, as shown in Figure 4. This suggests an additional confirmation of the formation of isostructural solids.

**Figure 5:**
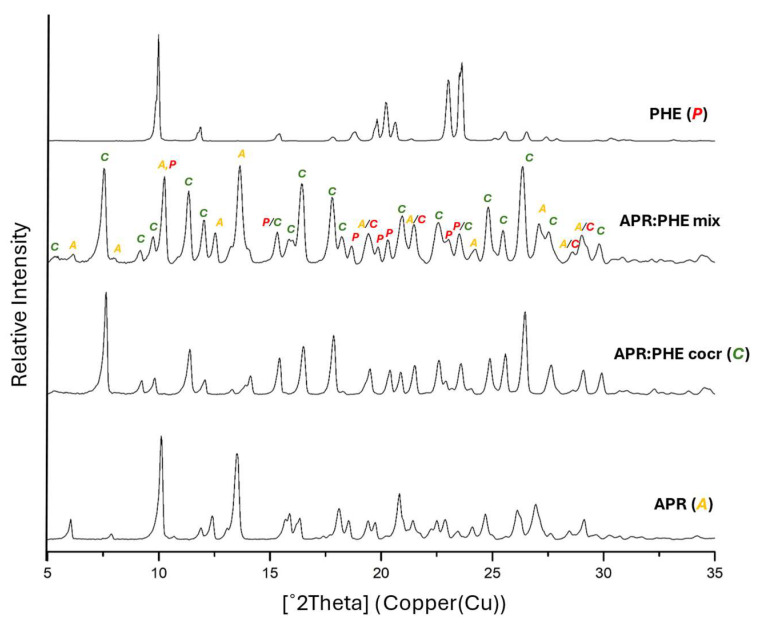
Comparison of the APR and PHE mixture, reagents PXRD patterns, with PXRD pattern of APR_PHE cocrystal in the range 5–30° 2*θ*. In the PXRD of the APR:PHE mixture, the letters indicate the peaks corresponding to phenol (*P*), Apremilast (*A*) and the APR:PHE cocrystal (*C*) formed in the mixture.

Next, the PXRD analysis of all APR mixtures with coformers was conducted to compare with newly obtained solid forms and to check the APR and coformers ability to react with each other in a mixture at ambient conditions. All mixtures were stable, except the mixture of APR with PHE. It was found that APR forms a solid form with phenol in the mixture at ambient temperature. The low melting point of phenol and its ability to absorb air moisture are attributed to the interaction between APR and PHE at ambient temperature. On the PXRD pattern (Figure 5), diffraction maxima belonging to APR:PHE are observed in the 7.4–11.4° 2*θ* range. Nevertheless, Apremilast did not entirely react with phenol, which can be evidenced by the presence of diffraction maxima of both Apremilast and phenol on the diffractogram of the mixture. Resembling behavior was not found in the rest cases. All PXRD patterns are in the Supporting Information File (Figure S2).

### 2.5. Physico-Chemical Properties

Modifying API crystal structures optimizes important physicochemical parameters. One important parameter is the thermal stability of the new solid forms of API, which can be obtained by thermal analysis.

**Figure 6:**
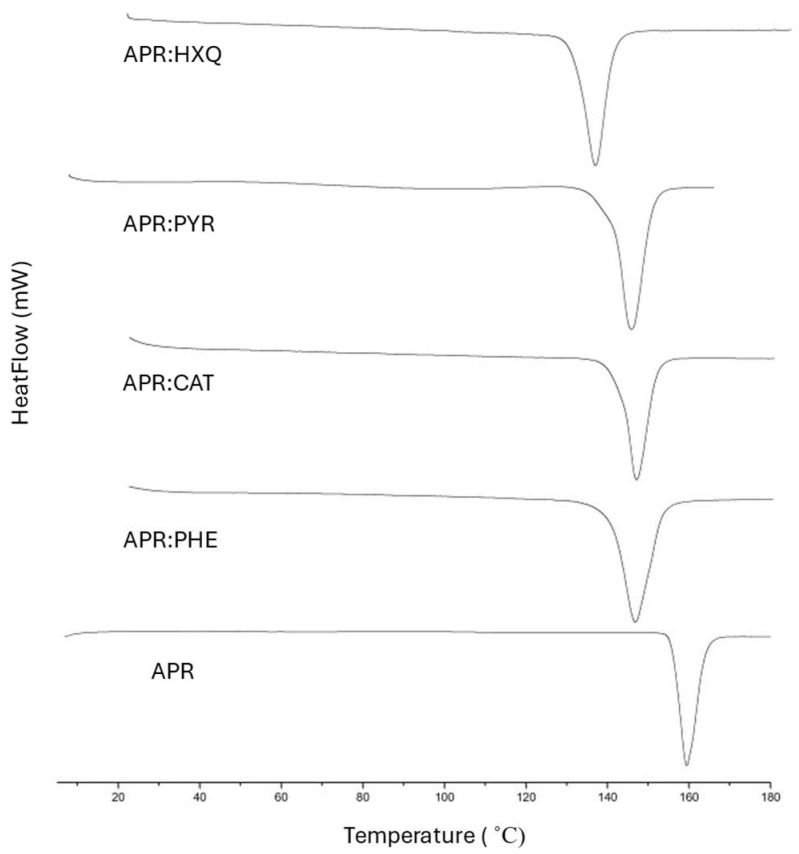
DSC thermograms of APR hemihydrate cocrystals with PHE, CAT, PYR and HXQ.

DSC is a widely used method of thermal analysis in the pharmaceutical industry. One endothermic peak at the melting point of the new APR forms confirms the purity of the samples and indicates the cocrystal stability in the range of 20–200 °C (Figure S1). The DSC of the APR solid forms is presented in Figure 6. The melting points of the starting materials and the new solid forms are shown in Table 2.

In general, the melting points of the hemihydrate cocrystals are lower by about 10 °C than the APR melting point. It seems likely that the number of intermolecular interactions between the coformer and API does not increase with the increasing number of hydroxyl groups in the benzene ring of coformer.

The melting point of the two isomers, APR:PYR and APR:HXQ, differ by about 15 °C. This difference can be related to the strength of hydrogen bond between APR, H_2_O and coformer as described above.

### 2.6. Hirshfield Surface Analysis

The structural description of Apremilast cocrystals and the thermal analysis results allow us to conclude that, despite the number and position of functional groups in the phenolic-type coformer, the number of hydrogen interactions remains unchanged. Hirshfeld surface analysis and the associated fingerprint plot (FP) method can reveal this aspect. Fingerprint plot (FPs) as a visual method allows us to quantitatively compare the intermolecular interactions in different structures.

The Hirshfeld surfaces and fingerprints for APR and coformer molecules for every structure are presented in the Supporting Information File Figure S3. Moreover, the fraction (percentage share) of each contact for each component in the four crystal structures is presented in Table 3.

Hirshfeld surfaces have been mapped over a *d_norm_* range (−0.5102–1.482 Å). The bright red spots on the surface show the strongest and shortest O-H⋯O and N-H⋯O bonds, whereas the light red spots are attributed to weak C-H⋯O hydrogen bonds. Hirshfeld surfaces are almost identical for APR molecules in every structure and reflect similar contacts.

**Figure 7:**
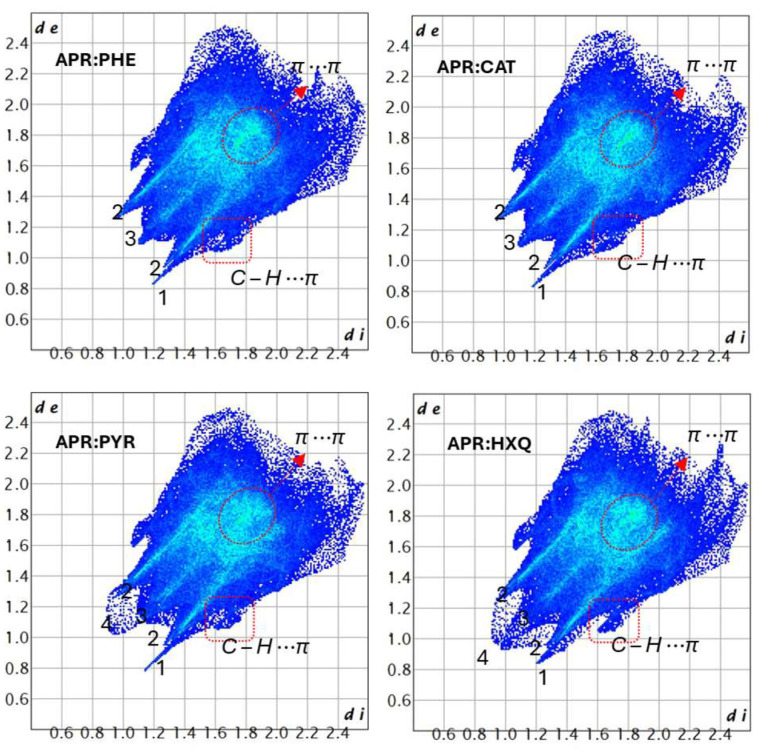
Corresponding two-dimensional fingerprint plots of APR:PHE, APR:CAT, APR:PYR, and APR:HXQ.

The FP plots for all crystals in Figure S3 show all contributions of interactions between atoms inside and outside the HS. The dominant intermolecular interactions within the molecular crystal packing can be attributed to H···H contacts (the broad spike labeled 3 in the FP plot (Figure 7) with contributions of 42.2%, 43.2%, 42.5%, and 41.7% for APR:PHE, APR:CAT, APR:PYR, and APR:HXQ, respectively), followed by hydrogen bonds (≈37%), C···C and C···H/H···C contacts (≈15%) for all APR molecules. The high contribution of H···H contacts appears due to the methyl groups present in the APR structure. In the FP plots of APR:PYR and APR:HXQ, the broad spikes at (*d_i_* + *d_e_* ≈ 1.9 Å) and (*d_i_* + *d_e_* ≈ 1.8 Å) labeled as 4 are indicative of H···H contacts between hydrogen atoms of the hydroxyl group of coformers and the methyl groups of APR.

The following significant fraction formed by OH contacts to the surface of APR. These contacts on the FP plots (Figure 7) are shown as two characteristic distinct and sharp spikes with less point distribution. The peak labeled 1 on the plot shows O···H/H···O contacts associated with O6··H481–O48 hydrogen bond, and the peak labeled 2 is assigned to O5··H161–C16 contact. However, the contribution of hydrogen bonds compared to the rest of the contacts is relatively small. The relatively low fraction of the strong hydrogen bonds contacts (O···H/H···O) to the Hirshfeld surface of APR can explain the low melting point of APR solid forms than the single APR.

Also, contacts C···H/H···C have a relatively significant share of the surface compared with other weak contacts. This fact confirms the presence of very weak C-H-π interactions. There are characteristic “wings” in the upper left and lower right parts of the FP (Figure 7), which are delineated as the result of C···H⋯π-interactions. The wings on the upper left (*d_i_* < *d_e_*) represent points on the surface around the C-H donor, and the wings on the lower right (*d_e_* > *d_i_*) belong to the surface around the π acceptor. Then, it can be noticed that APR acts as a π acceptor to a greater extent (C···H ≈ 9%) than as a C···H donor (H-C, ≈6%) while phenolic coformer appears to be more in role of a donor (C-H/H-C, 5.5–6.2%/8–12%). Dudek et al. [12] have found that aromatic coformers engaged in aromatic–aromatic interactions with APR molecule have electron–enriched rings and act like hydrogen bond donors. The significant contribution of C-H···O short contacts to the stabilization was observed as well. The contribution of these contacts to the surface of the HXQ coformer is lower in comparison with other guest molecules resulting in the change of thermal stability. Interestingly, C···C contacts, compared with other intermolecular contacts, share around 2.5% to the surface, despite the parallel arrangement of the aromatic rings.

FP plots of coformer structures are presented in Supporting Information File Figure S4 and contact fractions for each structure are in Supporting Information File Table S2. Crystal structures for PHE (201623), CAT (185293), and PYR (924230) were downloaded from CSDC [4].

The table of contacts in the (Supporting Information File Table S2) and Hirshfeld surface plots (Supporting Information File Figure S4) shows that, in the series of pure coformers (PHE—CAT—PYR), the share of hydrogen bonds increases with an increasing number of hydroxyl groups. The sharp peaks on the FP plot of PYR correspond to hydrogen bonds highlighted in a brighter green color compared to the FP plot of CAT). The share of C-H-π interactions decreases (wings on the FP of PYR are practically absent), which is why their melting temperatures change (Table 2). In contrast, the fraction of both hydrogen bonds and the fraction of other interactions are only slightly different in APR cocrystals, which is in relation with low fluctuation of their melting temperatures (Table 2).

## 3. Materials and Methods

### 3.1. Materials

Apremilast form B (APR) [N-[2-[(1S)-1-(3-ethoxy-4-methoxyphenyl)-2-methylsulfonylethyl]-1,3-dioxoisoindol-4-yl]acetamide was provided by the pharmaceutical company Zentiva, Prague, Czech Republic. All used phenolic compounds and solvents were obtained commercially and utilized without additional purification). Phenol, p.a. (PHE) was purchased from Penta Co, Ltd., Prague, Czech Republic. Catechol, 99% (CAT) and Pyrogallol, 98% + (PYR) were obtained from ThermoFisher Scientific Co., Ltd., Brno, Czech Republic. Hydroxyquinol (HXQ), for synthesis, was purchased from Merck Co, Ltd., Prague, Czech Republic. The solvent (acetone) used in this study was purchased from Lach-Ner Co., Ltd., Neratovice, Czech Republic.

### 3.2. Preparation of Cocrystals

To obtain APR cocrystals with PYR and CAT, respectively, Apremilast and coformers were taken in a respective molar ratio (2:1). Various methods have been employed to obtain cocrystals: slow evaporation from solution, mortar-pestle grinding method, and the grinding using the planetary ball mill. Attempts to obtain cocrystals using different methods are presented in Table 4. A total of 2–3 drops of water were added to the mixture of substances in each method. Planetary ball mill (PM 100, Retsch, Haan, Germany) equipped with a 50 mL zirconium oxide jar and a 5 mm ball was used for the grinding method. The grinding process was carried out for a total of 60 min at a speed of 400 rpm. Five cycles of 15 min grinding, then a 5 min pause, and 5 cycles were used. After each cycle, the mixture was allowed to cool for 5 min before reversing the direction of rotation. To synthesize PHE: APR solid forms, Apremilast and PHE were mixed in a 2:1 molar ratio and ground with a mortar and pestle along with 2–3 drops of water. The resulting mixtures of APR: PYR, APR: PHE, and APR: CAT were dissolved in acetone and then evaporated to obtain a suitable single crystal for SCXRD. APR: HXQ cocrystals were formed by dissolving Apremilast and HXQ in a 2:1 molar ratio using acetone as a solvent for the slow evaporation method. Acetone as a solvent was chosen for its excellent solubility of Apremilast and coformers, and to prevent the creation of Apremilast solvates with solvents. Different attempts to obtain new phases are presented in Table 4. Once formed, the cocrystals were dried under normal conditions and further characterized via SCXRD, PXRD, DSC. The structure of HXQ is not known. All attempts to prepare single-crystals suitable for SCXRD have failed.

### 3.3. Single Crystal X-Ray Diffraction (SCXRD)

SCXRD data of APR:PHE, APR:CAT, APR:PYR, APR:HXQ cocrystals were collected on the Bruker D8 VENTURE system equipped with charge-integrating pixel array detector Photon II 7 (Bruker AXS GmbH, Karlsruhe, Germany), a multilayer monochromator and a CuKα Incoatec microfocus sealed tube (λ = 1.54178 Å) using combined φ and ω scans at 180(2) K. Diffraction data collection, Unit Cell refinement, and data reduction were performed using the APEX 4 program [26].

The APR:PHE and APR:HXQ structures were solved by SHELXT [27]. The remaining structures were solved by SIR92 [28] and refined with the Crystals package [29] by the full-matrix least-squares method on *F*^2^. All hydrogen atoms were located in the difference Fourier map. The hydrogen atoms attached to carbon atoms were placed geometrically with *U*_iso_(H) in the range of 1.2–1.5 *U*_eq_ of the parent atom and were refined with riding constraints [30]. The hydrogen atoms attached to hetero atoms in the structure of the coformer molecules were placed geometrically and refined at geometrically constrained riding positions. In APR:PHE, hydrogen atoms attached to the oxygen atom of the coformer were refined with soft restraints. For the structures APR:PYR and APR:HXQ the hydrogen atoms attached to hetero atoms in the structure of the coformer molecules were placed geometrically, and their coordinates were refined. During the final refinement, the structures were refined using a riding model due to unstable hydrogen position. The guest molecule was disordered over the two-fold axes with an occupancy of 0.5 for the hydroxyl groups in all structures. The crystallographic data for all structures were deposited at the Cambridge Crystallographic Data Center (CCDC 2393588-2393591).

The crystallographic data are summarized in Table 5. Selected hydrogen bonds and geometrical parameters are given in Table 1.

The MERCURY package (Mercury 2022.2.0 (Build 353591)) was used to analyze intermolecular contacts in the crystal structures of new APR solid forms [31].

### 3.4. Powder X-Ray Diffraction (PXRD)

X-Ray powder diffraction data were collected at room temperature with an X’Pert^3^ Powder *θ*-*θ* powder diffractometer with parafocusing Bragg–Brentano geometry using CuKα radiation (*λ* = 1.5418 Å, *U* = 40 kV, *I* = 30 mA) (Malvern Panalytical, Malvern, UK). An ultrafast detector PIXCEL (Malvern Panalytical, Malvern, UK) was employed to collect XRD data over the angular range from 5 to 50° (2*θ*) with a step size of 0.039° 2*θ*, and a counting time of 0.706 s/step. Data evaluations were performed in the software package HighScore Plus 3.0. [32]

### 3.5. Differential Scanning Calorimetry (DSC)

The differential scanning calorimetry (DSC) curves were recorded employing the DSC 131 (Setaram, Caluire-et-Cuire, France). The heating rate was 10 °C/min in the temperature range of 5–250 °C. APR:PHE, APR:CAT, APR:PYR, APR:HXQ, and APR samples were weighed at about 10 mg and placed in the aluminum pans with a lid.

### 3.6. Hirshfield Surface

Crystal Explorer 21.5 [33] was used to analyze Hirshfeld Surface 34] and 2D fingerprint (FP) plots [34] for the crystal structures. Hirshfeld surface was generated for APR forms and coformer structures and mapped using the *d_norm_* function. *d_norm_* function is the normalized sum of *d_e_* (distance from the point of surface to the nearest atom outside the surface) *d_i_* (distance from the point of surface to the nearest atom inside the surface) [33]. The two-dimensional fingerprint plot makes it possible visually to summarize the frequency of each combination of *d_e_* and *d_i_* over the whole molecular surface to estimate which intermolecular interactions are present and which area of surface corresponds to these interactions. The FPs were constructed in the range 0.0 < *d* < 2.5 Å. Points on the FPs are colored according to the share of the surface area. Points with no contribution are left uncolored, blue-colored points have the smallest contribution, and points with greater contribution are colored with red [35].

## 4. Conclusions

Four novel solid forms of Apremilast (APR) were synthesized by cocrystallizing with a series of phenolic compounds and characterized using various analytical techniques. These forms represent a valuable series of hydrated APR cocrystals, where the composition and stability can be considered as chemical variables. Contrary to expectations, the variability in molecular packing did not change with the increasing number or variety of hydroxyl groups in the coformers. The broad range of coformer melting points reflects their ability to form strong hydrogen bond networks; however, surprisingly, these networks are not utilized in the cocrystal molecular packing. All cocrystals crystallize in the same space group *P*4_1_2_1_2, and they adopt the same structural type, indicating analogous both the conformation and the molecular packing. The absence of a complex hydrogen-bonding network likely facilitates rotational disorder in the coformer molecules, which was observed in all of the structures. Hirshfeld surface analysis further supported the structural relationship among the presented forms, revealing limited use of hydrogen bond donors and acceptors. The melting points of the new forms were found to be close to that of APR, with the narrow range of values consistent with the restricted hydrogen bond network.

## Figures and Tables

**Table 1:** Important hydrogen-bonding distances and parameters of the APR hemihydrate cocrystal.

Crystal Structure	APR:PHE	APR:CAT	APR:PYR	APR:HXQ
*d*, (O47···O50), Å	2.713(4)	2.609(4)	2676(4)	2.515(6)
*<*(O47–H··· O50), ˚	166(5)	148	169	163
*d*, (O50···O6), Å	2.993(2)	2.929(3)	2.883(3)	2.952(4)
*<*(O50–H··· O6), ˚	165(4)	156	160	159
*d*, (π–π) Å	4.1187(11)	4.1301(19)	4.1255(17)	4.127(2)
*<*(π–π), ˚	5.35(9)	4.81(15)	4.40(13)	5.81(18)

**Table 2:** Melting points for starting materials and new solid forms.

API, Melting Point (°C)	Coformer, Melting Point (°C)	Solid Form, Melting Point (°C)
APR (156)	PHE (40) [22]	APR:PHE (145)
	CAT (110) [23]	APR:CAT (141)
	PYR (126) [24]	APR:PYR (142)
	HXQ (139) [25]	APR:HXQ (127)

**Table 3:** Fraction of each contact in the environment for each component in the crystals.

Contacts	APR:PHE	APR:CAT	APR:PYR	APR:HXQ
	APR			
C···C, %	2.4	2.6	2.5	2.5
C···H/H···C, %	9.2/6.2 (15.4)	9.3/6.0 (15.3)	8.8/6.0 (14.8)	8.3/6.1 (14.4)
C···O/O···C, %	1/0.4 (1.4)	0.7/0.4 (1.1)	1.3/0.4 (1.7)	1.9/0.4 (2.3)
C···N/N···C, %	0.4/0.8 (1.2)	0.4/0.8 (1.2)	0.3/0.8 (1.1)	0.4/0.8 (1.2)
O···N/N···O, %	-	-	0/0.2	0/0.3
O···O, %	0.1	0.1	0.1	0.1
H···H, %	42.1	43.2	42.5	41.7
N···H/H···N, %	0.3/0.1 (0.4)	0.3/0.1 (0.4)	0.1/0.1 (0.2)	0.1/0.1 (0.2)
O···H/H···O, %	16.9/20.1 (37.0)	15.7/20.4 (36.1)	16.5/20.4 (36.9)	17.1/20.2 (37.3)
**Coformer**	**PHE**	**CAT**	**PYR**	**HXQ**
C···C, %	8.3	8.5	8.1	8.4
C···H/H···C, %	6.1/11 (17.1)	6.0/12.5 (18.5)	5.5/10.9 (16.4)	6.0/8.2 (14.2)
C···O/O···C, %	1.0/4.1 (5.1)	0.7/2.1 (2.8)	0.8/4.7 (5.5)	0.7/7.9 (8.6)
C···N/N···C, %	2.7/0 (2.7)	2.7/0 (2.7)	2.7/0 (2.7)	2.7/0 (2.7)
O···N/N···C, %	-	-	1.2/0 (1.2)	1.9/0 (1.9)
O···O, %	0.2	0.5	0.1	-
H···H, %	38.6	52.0	43.2	34.9
N···H/H···N, %	0/1.7 (1.7)	0/1.6 (1.6)	0/0.5 (0.6)	-
O···H/H···O, %	13.0/13.3 (26.3)	11.9/1.5 (13.4)	13.3/8.9 (22.2)	16.9/12.4 (29.3)
**Water**	**H_2_O**	**H_2_O**	**H_2_O**	**H_2_O**
H···H, %	38.8	40.1	41.1	46.8
O···H/H···O, %	28.3/32.9 (61.2)	26.2/33.7 (59.9)	27.0/31.9 (58.9)	24.7/28.5 (53.2)

**Table 4:** Results of the used methods to obtain cocrystals.

	Slow Evaporation from Solution	Mortar-Pestle Grinding Method	Grinding Using the Planetary Ball Mill
APR:PHE	✗	✓	✗*
APR:CAT	✗	✗	✓
APR:PYR	✗	✓	✓
APR:HXQ	✓	✓	✓

✓—new solid forms were successfully obtained, ✗—product with both APR and coformers. ✗*—the grinding method using the planetary ball mill was not used for cocrystals due to toxicity of phenol.

**Table 5:** Crystallographic data and refinement details for the described Apremilast solid forms (systems).

	APR:PHE·H_2_O	APR:CAT·H_2_O	APR:PYR·H_2_O	APR:HXQ
CSD No.	2,393,590	2,393,588	2,393,591	2,393,589
Molecular Formula	(C_22_H_24_N_2_O_7_S) 0.5(C_6_H_6_O)0.5(H_2_O)	(C_22_H_24_N_2_O_7_S) 0.5(C_6_H_6_O_2_)0.5(H_2_O)	(C_22_H_24_N_2_O_7_S) 0.5(C_6_H_6_O_3_)0.5(H_2_O)	(C_22_H_24_N_2_O_7_S) 0.5(C_6_H_6_O_3_)0.5(H_2_O)
*M_r_*, g mol^−1^	516.82	524.57	531.56	524.57
Crystal system	Tetragonal	Tetragonal	Tetragonal	Tetragonal
Space group	*P*4_1_2_1_2	*P*4_1_2_1_2	*P*4_1_2_1_2	*P*4_1_2_1_2
*a*, Å	12.9102(10)	12.91040(14)	12.8890(2)	12.8833(2)
*c*, Å	29.6214(6)	29.6743(6)	29.7498(8)	29.9010(5)
*V*, Å^3^	4937.10(13)	4946.07(15)	4942.2(2)	4962.95(18)
*Z*	8	8	8	8
*ρ_calc_*, g/cm^3^	1.390	1.409	1.431	1.404
*F_000_*	2176	2208	2232	2228
*µ_CuKα_*, mm^−1^	1.623	1.644	1.670	1.663
*T*, K	180	180	180	180
Crystal size, mm^3^	0.080 × 0.110 × 0.171	0.200 × 0.202 × 0.380	0.200 × 0.228 × 0.240	0.074 × 0.076 × 0.136
*θ* range, °	3.73–72.22	3.73–70.07	0.97–72.16	3.74–68.27
Data collected	87,075	68,679	69,008	62,462
Data obs., *I* > 2 *σ*(*I*)	4777	4700	4911	4562
Data/Restrains/*N_par_*	4866/9/344	4700/3/334	4911/45/343	4562/67/342
*R*_1_ (*I* > 2 *σ*(*I*))	0.0293	0.0386	0.0338	0.0429
*wR*_1_ (*I* > 2 *σ*(*I*))	0.0808	0.1125	0.0930	0.1149
*R*	0.0298	0.0389	0.0346	0.0446
*wR*	0.0815	0.1136	0.0938	0.1173
GOF	0.99	0.99	0.99	1.00
Δ*ρ_min_*, Δ*ρ_max_*, e Å^−3^	−0.21, 0.16	−0.37, 0.78	−0.35, 0.24	−0.63, 0.34
Flack’s *x*	0.009(12)	0.001(19)	0.002(15)	0.007(15)

## Data Availability

The data of the current study are available from the corresponding authors on reasonable request.

## Supplementary Materials

The following supporting information can be downloaded at: https://www.mdpi.com/article/10.3390/molecules29246060/s1, Hydrogen-bonding distances and parameters of the APR hemihydrate cocrystal (Table S1), DSC data (Figure S1), PXRD patterns (Figure S2), Fingerprint plots (Figure S3), Fingerprint plots for coformers crystal structure (Figure S4), Fraction of contacts for coformers crystal structures (Table S2).
