# Supplementary material for: Apremilast Cocrystals with Phenolic Coformers
Source: Molecules. 2024 Dec 23;29(24):6060. doi: 10.3390/molecules29246060 (PMC11678279; doi:10.3390/molecules29246060)
Supplement: Supplementary file 1 [file molecules-29-06060-s001.zip › molecules-3318873-supplementary.pdf]

Supplementary Materials

Apremilast Cocrystals with Phenolic Coformers

Yelizaveta Naumkina [1,2], Bohumil Kratochvíl [1], Elena Korotkova [2], Jan Čejka [1,*]

[1] Department of Solid State Chemistry, University of Chemistry and Technology, Prague, Technicka 5, 16628 Prague, Czech Republic; naumkiny@vscht.cz (Y.N.); bohumil.kratochvil@vscht.cz (B.K.)
[2] Chemical Engineering Department, National Research Tomsk Polytechnic University, Lenin Avenue 30, 634050 Tomsk, Russia; eikor@tpu.ru
* Correspondence: jan.cejka@vscht.cz; Tel.: +420-220-44-4200

Table of Contents

1. List of the hydrogen bond distances 2

2. DSC curves of APR cocrystals 4

3. PXRD patterns of APR coformer systems 6

4. Hirshfield fingerprint plots 8

1. List of hydrogen bond distances

Table S1. Hydrogen bonding distances and parameters of the APR hemihydrate cocrystal (D: donor; A: acceptor)

APR:	D – H ···A	*d*(D – H), Å	*d*(H··· A), Å	*d*(D ···A),Å	<(D – H··· A), °
PHE	N2 – H22···O1	0.84(18)	2.286(16)	2.9657(17)	137.8(14)
	O47 – H471···O50	0.84(5)	1.89(6)	2.713(4)	166(5)
	O50 – H481···O6	0.856(17)	2.157(18)	2.993(2)	165(4)
	C2 – H21···O3	0.95	2.29	2.901(2)	122
	C11 – H111···O1	0.98	2.51	2.9175(18)	105
	C12 – H121···O2 [#1]	0.99	2.32	3.1651(19)	142
	C12 – H122···O3[#2]	0.99	2.50	3.457(2)	162
	C13 – H131···O1 [#3]	0.96	2.56	3.052(2)	112
	C15 – H151···O4[#2]	0.97	2.47	3.299 (2)	143
	C16 – H16 ···O5[#2]	1.02	2.40	3.384(2)	161
	C19 – H191···O2 [#1]	0.89	2.44	3.3349(19)	178
	C20 – H201···O3 [#4]	0.95	2.55	3.483(3)	167
	C43 – H43 ···O4 [#5]	0.93	2.56	3.480 (1)	172
Symmetry code: [1#] *y*,*x*,1-*z*; [2#] 1/2+*x*,1/2-*y*,3/4-*z*; [3#] 1/2-*y*,-1/2+*x*,1/4+*z*; [4#] 3/2-*y*,1/2+*x*,1/4+*z*; [5#] 1+*y*,1+*x*,1-*z*					
CAT	N2 – H21···O1	0.85	2.28	2.968(2)	139
	O47–H362···O50 [#5]	0.90	1.81	2.609(4)	148
	O50– H372···O6 [#6]	0.84	2.14	2.929(3)	156
	C2 – H210 ···O3	0.96	2.58	3.496(3)	158
	C11 – H111···O1	0.98	2.46	2.920(3)	108
	C12 – H121···O3 [#2]	0.97	2.50	3.455(3)	166
	C12 – H122···O2 [#1]	0.98	2.31	3.172(3)	146
	C13 – H131···O1 [#3]	0.97	2.40	3.050(3)	124
	C15 – H151···O4 [#2]	0.95	2.54	3.308(3)	138
	C16 – H181···O5 [#2]	0.95	2.47	3.385(3)	163
	C19 – H191···O2 [#1]	0.95	2.39	3.340(3)	176
	C20 – H203···O3 [#4]	0.95	2.58	3.494(3)	161
Symmetry code: [1#] *y*,*x*,1-*z*; [2#] ½ -*x*,1/2+*y*,5/4-*z*; [3#] -1/2+*y*,-1/2-*x*,-1/4+*z*; [4#] 1/2+*y*,3/2-*x*,-1/4+*z*; [5#] 3/2-*y*,1/2+*x*,1/4+*z*; [6#] 3/2-*x*,-1/2+*y*,5/4-*z*					
PYR	N2 – H21···O2	0.82	2.31	2.973(2)	139
	O47 – H471···O48	0.96	2.11	2.469(6)	100
	O47 – H472···O50[#]	0.94	1.75	2.676(4)	169
	O50 – H501···O6 [#5]	0.86	2.06	2.883(3)	160
	C11 – H···O2	0.98	2.49	2.9170(19)	106
	C12 – H121···O3 [#2]	0.97	2.31	3.166(2)	147
	C12 – H122···O1 [#1]	0.97	2.49	3.452(2)	173
	C13 – H131···O2 [#3]	0.96	2.48	3.049(3)	117
	C13 – H132···O4 [#2]	1.00	2.55	3.433(3)	147
	C15 – H151···O3 [#2]	0.91	2.46	3.360(2)	172
	C18 – H181···O5 [#1]	0.92	2.48	3.372(2)	164
	C19 – H191···O4 [#1]	0.92	2.56	3.305(2)	139
	C22 – 201···O1 [#4]	0.94	2.59	3.482(3)	158
	C2 – H210···O1	0.91	2.32	2.899(2)	121
	C21 – H213···O48 [#2]	0.94	2.34	3.252(6)	163
	C43– 351···O4 [#1]	0.97	2.51	3.443(3)	162

Symmetry code: 1# 3/2-*x*,1/2+*y*,5/4-*z*; 2# 1-*y*,1-*x*,3/2-*z*; 3# 3/2-*y*,-1/2+*x*,1/4+*z*; 4# 1/2-*y*,1/2+*x*,1/4+*z*; 5# -1/2+*y*,1/2-*x*,-1/4+*z*

HXQ	N2 – H21···O1	0.84	2.30	2.976(3)	138
	O50 – H501···O6 #4	0.84	2.15	2.952(4)	159
	O47 – H472···O50	0.97	1.57	2.515(6)	163
	C11 – H111···O1	0.97	2.46	2.914(3)	108
	C12 – H121···O2 #1	0.97	2.32	3.169(3)	145
	C12 – H121···O3 #2	0.97	2.49	3.453(3)	174
	C13 – H131···O1#3	0.98	2.48	3.047(4)	117
	C13 – H131···O4#1	0.98	2.58	3.443(4)	147
	C15 – H151···O4 #2	0.93	2.56	3.316(3)	138
	C16 – H181···O5 #2	0.91	2.49	3.374(3)	165
	C19 – H191···O2 #1	0.94	2.45	3.376(3)	172
	C2 – H210···O3	0.95	2.32	2.895(3)	121
	C22 – H221···O48 #1	0.95	2.34	3.257(6)	163
	C43 – 331···O4 #2	0.97	2.50	3.440(4)	162

Symmetry code: 1# 1-*y*,1-*x*,3/2-*z*; 2# 3/2-*x*,1/2+*y*,5/4-*z*; 3# 3/2-*y*,-1/2+*x*,1/4+*z*; 4# 1/2-*x*,-1/2+*y*,5/4-*z*

2. DSC curves of APR cocrystals

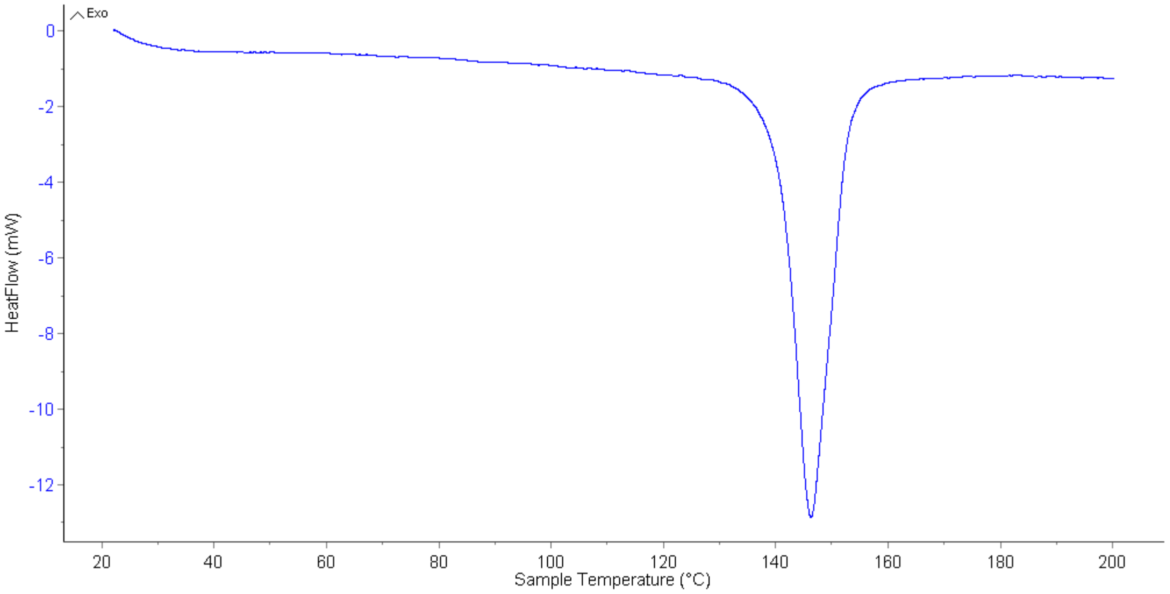

a) APR:PHE

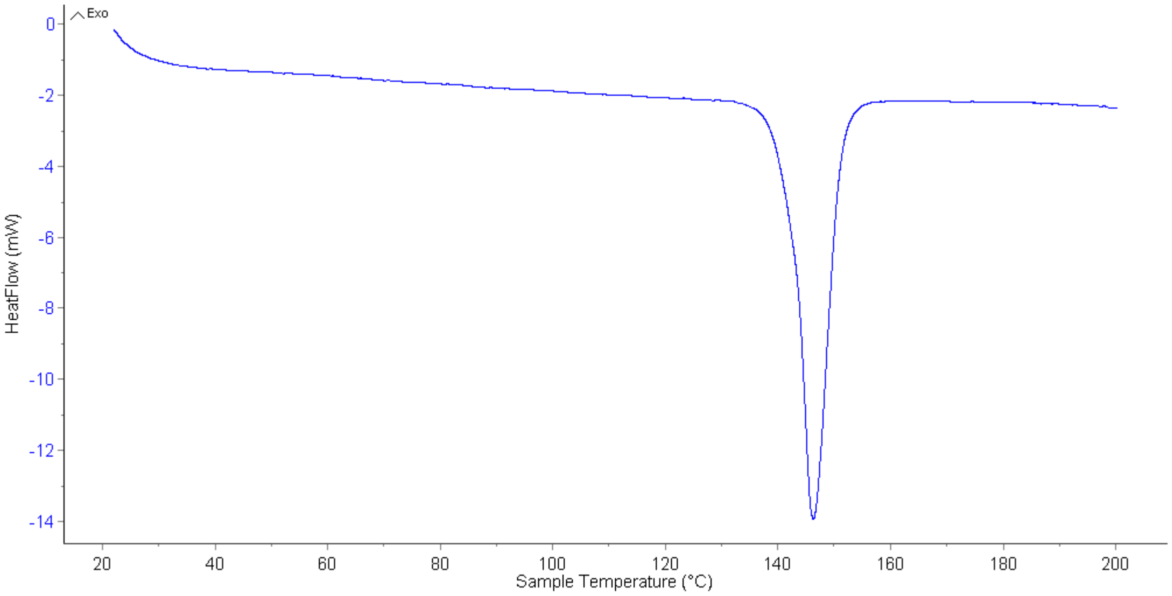

b) APR:CAT

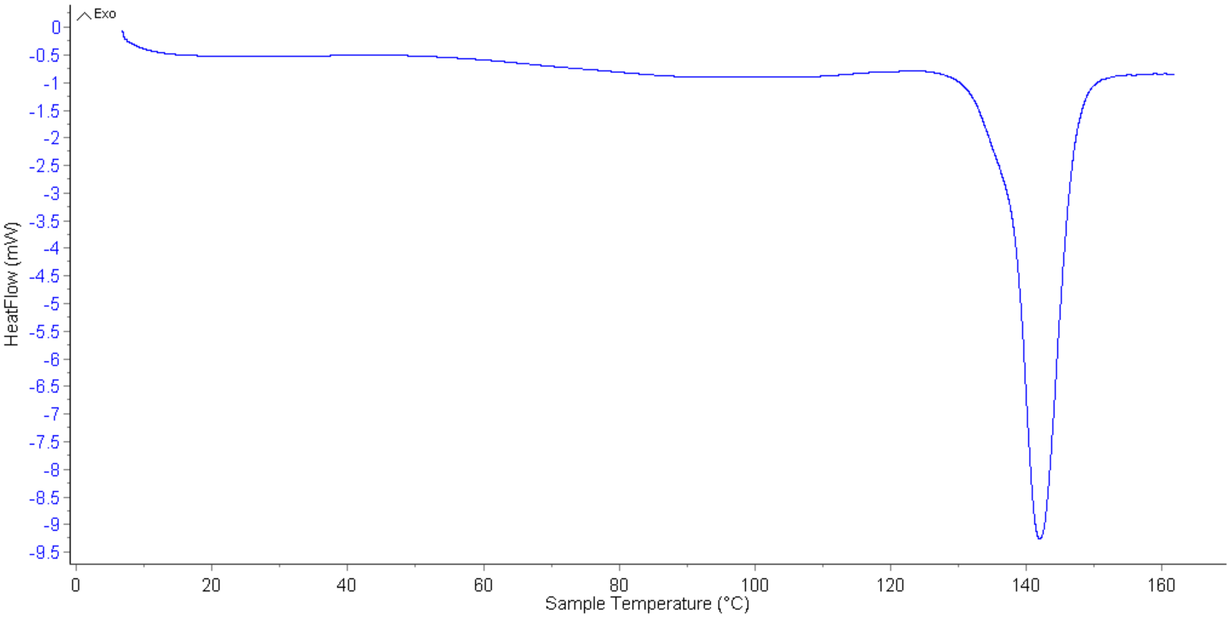

c) APR:PYR

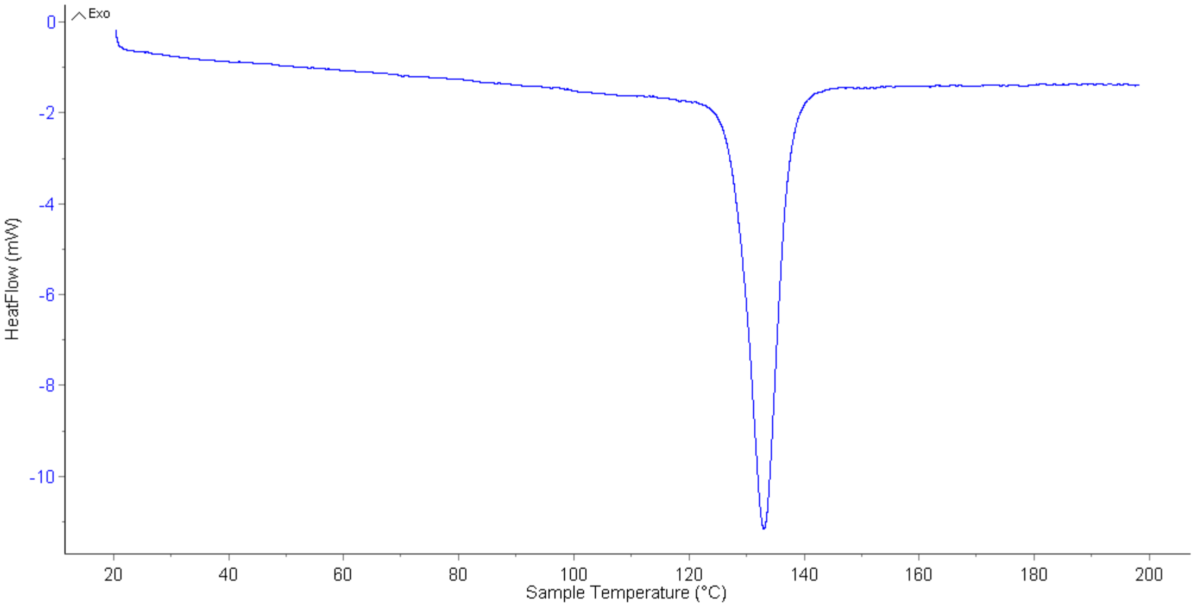

d) APR:HXQ

Figure S1. DSC curves of all presented multicomponent forms: a) APR:PHE, b) APR:CAT, c) APR:PYR (stopped at 165 °C, to prevent leaking out of pan), d) APR:HXQ

3. PXRD patterns of APR coformer systems

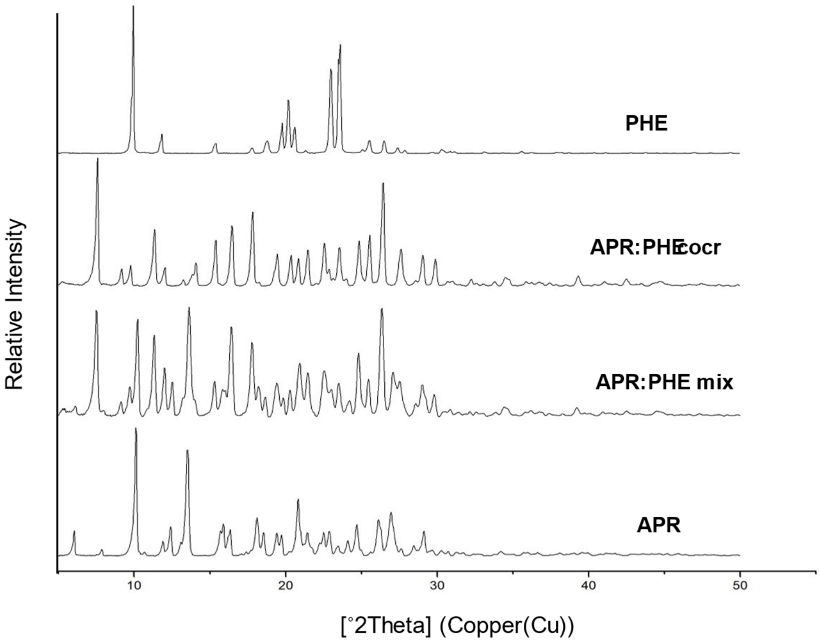

Figure S2 a) PXRD of APR:PHE system

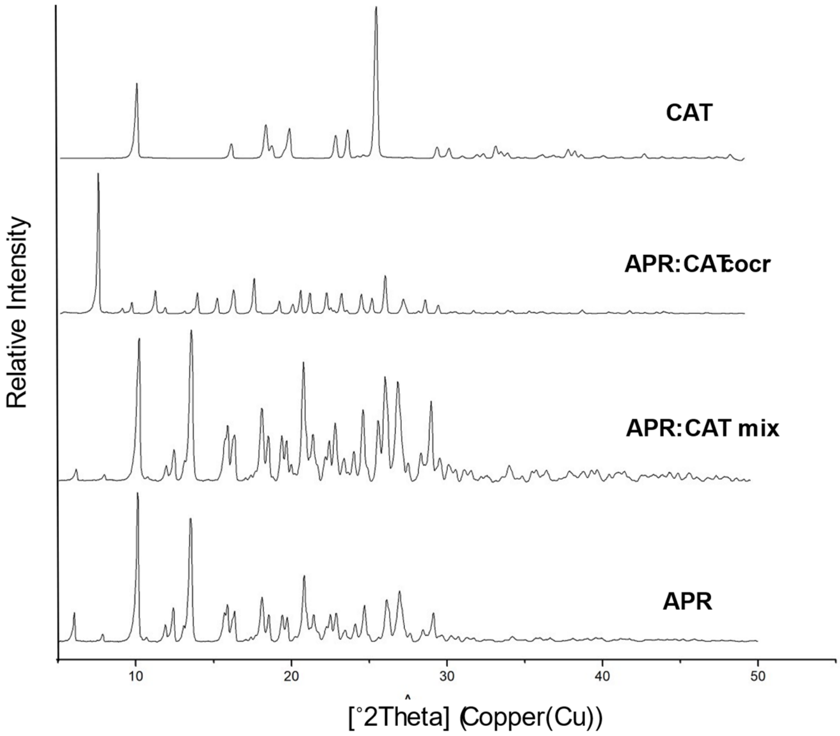

Figure S2 b) PXRD of APR:CAT system

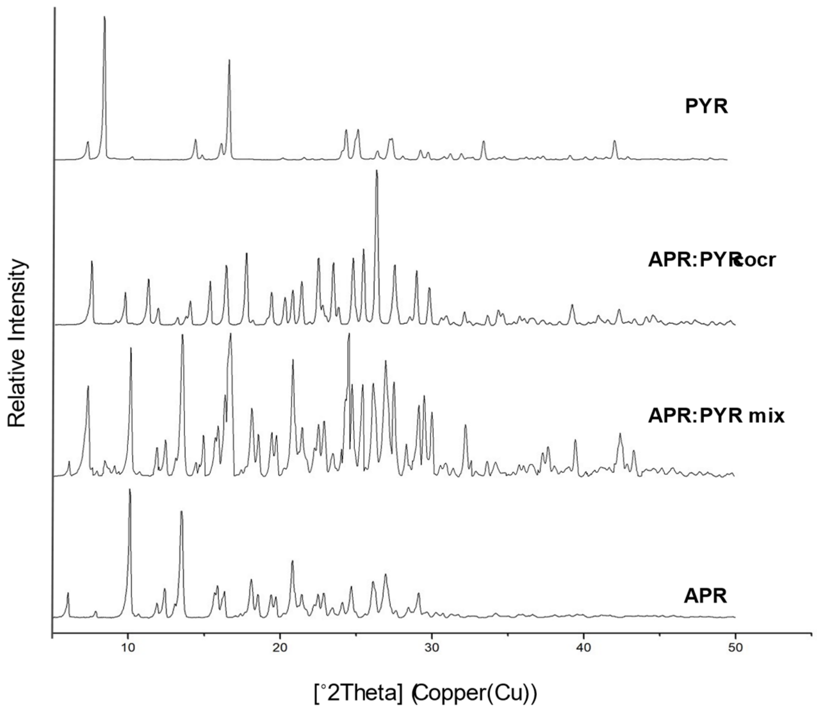

Figure S2 c) PXRD of APR:PYR system

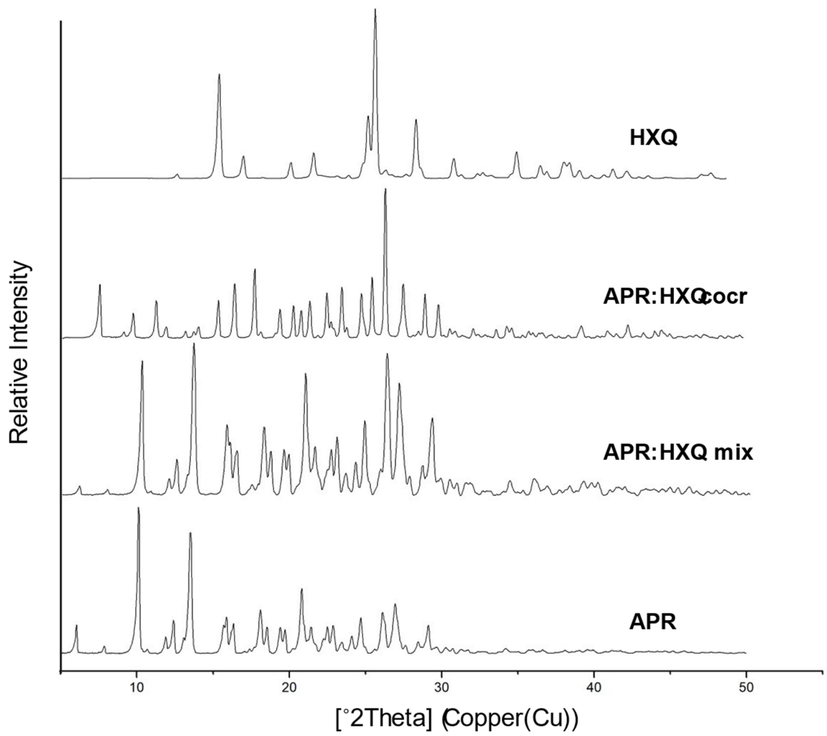

Figure S2 d) PXRD of APR:HXQ system

Figure S2. Comparison of the PXRD patterns of the reagents, cocrystals, and APR mixture with coformers.

4. Hirshfield fingerprint plots

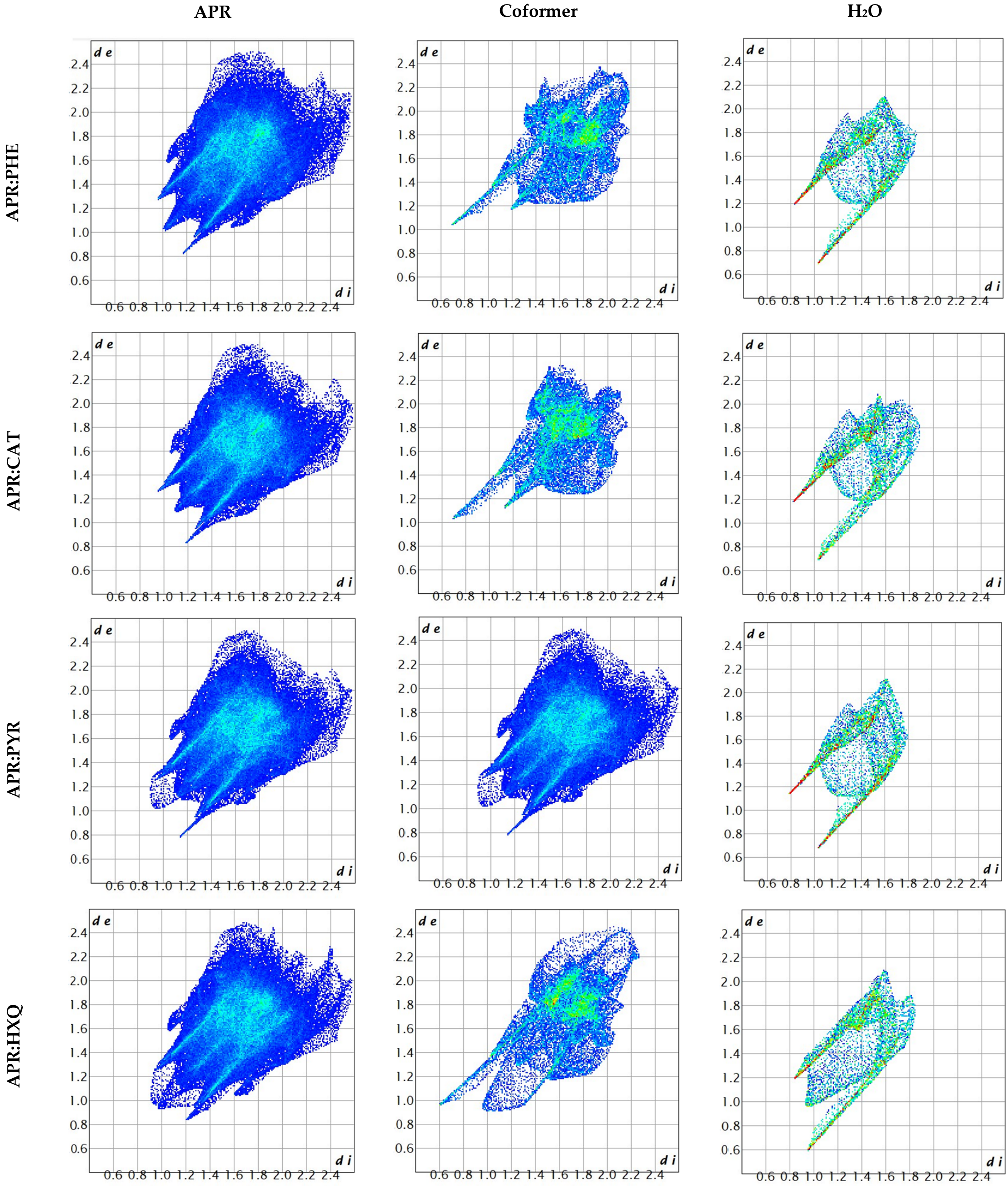

Figure S3. The fingerprints plots for APR, coformer and water for every cocrystal.

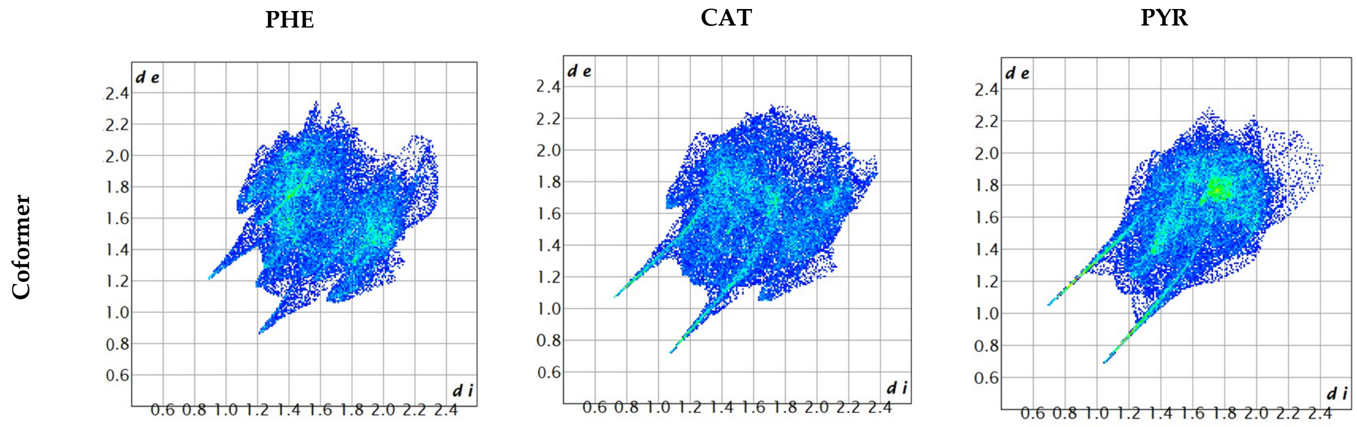

Figure S4. The fingerprints plots for coformers crystal structures.

Table S2. Fraction of contacts for coformers crystal structures.

Contacts	PHE	CAT	PYR
C···C, %	0	1.3	9.5
C···H/ H···C, %	20.6/14.4(35)	16.8/10.6(27.4)	7.8/5.0(12.8)
C···O/ O···C, %	0	2.4/1.8(4.2)	2.1/1.8(3.9)
O···O, %	0	0	1.9
H···H, %	44.9	42.3	40.8
O···H/ H···O, %	9.5/10.6(20.1)	13.5/11.3(24.8)	17.5/13.6(31.1)
